# An Expanded Agenda for the Primary Prevention of Breast Cancer: Charting a Course for the Future

**DOI:** 10.3390/ijerph17030714

**Published:** 2020-01-22

**Authors:** Mary C. White, Marion (Mhel) H. E. Kavanaugh-Lynch, Shauntay Davis-Patterson, Nancy Buermeyer

**Affiliations:** 1 Division of Cancer Prevention and Control, Centers for Disease Control and Prevention, Atlanta, GA 30341, USA; 2 California Breast Cancer Research Program, University of California, Oakland, CA 94612, USA; Marion.Kavanaugh-Lynch@ucop.edu; 3 Comprehensive Cancer Control Program, California Department of Public Health, Sacramento, CA 95816, USA; shauntay.davis@cdph.ca.gov; 4 Breast Cancer Prevention Partners, San Francisco, CA 94109, USA; nancy@bcpp.org

**Keywords:** breast cancer, California, cancer plan, comprehensive cancer control, environmental exposure, incidence, intervention, policy, primary prevention, risk factor

## Abstract

Advances in breast cancer science, early detection, and treatment have resulted in improvements in breast cancer survival but not in breast cancer incidence. After skin cancer, breast cancer is the most common cancer diagnosis in the United States. Each year, nearly a quarter million U.S. women receive a breast cancer diagnosis, and the number continues to rise each year with the growth in the population of older women. Although much remains to be understood about breast cancer origins and prevention, action can be taken on the existing scientific knowledge to address the systemic factors that drive breast cancer risk at the population level. The California Breast Cancer Research Program funded a team at Breast Cancer Prevention Partners (BCPP) to convene leaders in advocacy, policy, and research related to breast cancer prevention from across the state of California. The objective was the development of a strategic plan to direct collective efforts toward specific and measurable objectives to reduce the incidence of breast cancer. The structured, innovative approach used by BCPP to integrate scientific evidence with community perspectives provides a model for other states to consider, to potentially change the future trajectory of breast cancer incidence in the United States.

## 1. Introduction

For many women and their health care providers, breast cancer prevention is synonymous with mammography screening. Regular breast cancer screening of asymptomatic women through mammography is considered a form of secondary prevention, because the early detection of breast cancer through screening can lead to more effective treatment, better outcomes, and fewer deaths [1]. The decline in breast cancer mortality from 1975 to 2000 was attributed, in part, to mammography screening [2], although improvements in treatment likely drove most of that and subsequent declines [3,4]. Nearly 90% of the women diagnosed with breast cancer in the United States will survive at least 5 years [5]. Unlike vaccines, however, mammograms offer no protection against the onset of disease and are of potential benefit only for women who already have breast cancer. Just as the World Health Organization has defined health as more than the absence of disease, breast cancer prevention is more than the absence of deaths from breast cancer. The primary prevention of breast cancer, reducing cancer incidence, requires addressing the multitude of factors that drive breast tumor initiation and development. This article describes a structured, innovative approach to integrate scientific evidence with community perspectives to develop a plan to reduce the incidence of breast cancer in California.

## 2. Breast Cancer Etiology and Risk Factors

Breast cancer is a complex disease, with multiple biological tumor subtypes [6]. Despite decades of intensive research, many questions remain regarding the causes and basic biology of breast cancer. Most breast cancers are believed to arise from a combination of factors over many years [7,8]. Intrinsic risk factors for breast cancer taken together likely account for a limited percentage of the disease [9]. Research spanning several decades has uncovered a variety of other known and suspected risk factors for breast cancer, including physical inactivity, exposure to chemicals and radiation, and social and economic inequities, among others [7,8]. The proportion of breast cancer cases attributable to preventable risk factors has been estimated at nearly half or more [10,11,12,13]. Others question the interpretation of such estimates, given the multifactorial nature of cancer causation and realistic expectations for risk reduction [14,15,16]. For any individual case of breast cancer, we cannot pinpoint what caused the illness. Although much remains to be understood about breast cancer origins and prevention, action can be taken on the considerable body of scientific research that already exists.

For breast cancer, where a woman lives is related to her risk of being diagnosed with this disease. Worldwide, incidence rates are highest in Australia/New Zealand, Western, Northern and Southern Europe, and North America [17]. Previous research showed that when women migrated from Asia where breast cancer incidence was low to the United States, incidence rates of breast cancer were higher in the Asian immigrants and higher still in their daughters [18]. Similar patterns have been observed in multiple generations of Hispanic women and other studies of migrant women [19,20]. The fact that migration from one country to another can dramatically impact breast cancer incidence demonstrates the power that the physical and social environment can have on breast cancer development.

Some of the known risk factors for breast cancer can be modified by individual behavior to lower risk, and others require societal or systemic changes. Often, change can occur at multiple levels and involve both individuals and their environments. For behaviors characterized as “lifestyle,” such as consumption of alcoholic beverages and physical activity, social and environmental factors can influence behavioral choices, consistent with a socio-ecologic framework [21,22]. The label lifestyle connotes individual choice and arguably puts the focus on personal responsibility rather than on social and environmental circumstances [23]. Both context and personal agency can be addressed. Some unhealthy behaviors might be amenable to strategies aimed at both individuals (such as informing consumers), and societal changes (such as removing unsafe and unhealthy products from the marketplace) [24].

Breast cancer risk factors related to patient characteristics, such as age, race, and ethnicity, have been regarded as nonmodifiable [25]. However, these and other personal characteristics have social dimensions that may be amenable to intervention at the community level. For example, the sequential process of aging cannot be changed, but the negative societal influences that contribute to ageism and accelerated aging are not immutable [26,27]. Similarly, a person’s race has been shown to be related to social and economic disadvantage that drive cancer disparities [28]. Actions can be taken at a societal level to mitigate the negative impact of racism and social injustice [29,30].

Other authors have pointed to the importance of addressing breast cancer primary prevention through structural or systemic level interventions [31,32], but this approach has not become widespread in cancer prevention efforts. Many far-reaching primary prevention strategies suggest the development of population-level interventions to address issues beyond an individual’s control. These intervention strategies, for instance, can establish healthy communities through zoning or urban planning to re-engineer the built environment, including repaving or establishing sidewalks and bike trails, to support greater physical activity. Green chemistry approaches to develop and promote safer alternatives to chemical carcinogens in the workplace and consumer products are other examples of primary prevention. The evidence continues to grow, identifying potential environmental links to breast cancer [24,33,34,35,36], and these links deserve prominence in any primary prevention effort. 

As research and advocacy continue to expand collective understanding about risk and protective factors associated with breast cancer, opportunities to prevent breast cancer also continue to expand. The California Breast Cancer Research Program “paradigm project” illustrates a framework for viewing breast cancer as a complex disease through inclusion of all genetic, biologic, behavioral, environmental, and social factors into one model [37]. This framework emphasizes transdisciplinary methods that incorporate comprehensive approaches to primary prevention.

## 3. Trends in Breast Cancer Incidence in the United States

For primary cancer prevention at the population level, the outcome of interest is incidence rates. In the United States, breast cancer is a reportable disease, and a system of central cancer registries provides high quality data on incidence for all newly diagnosed breast cancers in this country [38]. Today, this system covers 100% of the U.S. population (https://www.cdc.gov/cancer/uscs/about/fact-sheet.htm. Each year, about 250,000 women and more than 2000 men receive a breast cancer diagnosis in the United States [39].

After skin cancer, female breast cancer is the most common type of cancer diagnosed among adults in the United States, surpassing the number of lung cancer cases diagnosed in men and women combined [40]. More than half of all new cases are diagnosed among women aged <65 years [5]. In 2016, 10.8% of all new breast cancer cases were diagnosed in California [40].

For most of the 20th century, beginning at least as early as the 1930s, breast cancer incidence increased in the United States [41]. From 1973 to 1998, the incidence rate for breast cancer increased more than 40% [42]. In the early 2000s, a small decline occurred in incidence, but not to previous levels [43]. Increases in incidence at the end of the last century were attributed, at least in part, to concordant trends in mammography [44]. The small drop in the early 2000s was linked to a decline in the use of hormone replacement therapy [45,46]. In recent years, breast cancer incidence has continued to rise, but less steeply [47]. The proportion of women up-to-date with mammography, about 72%, has not changed for over a decade [48].

Because of the continued aging of the population, the absolute number of breast cancer cases diagnosed each year is projected to increase, even if incidence rates remain unchanged [49]. Advances in breast cancer science, early detection, and treatment have reduced death rates for breast cancer but not halted the rise in the number of women diagnosed with this disease [5,50,51].

In 2016, an estimated 3.5 million women in the United States were living with a previous diagnosis of breast cancer, and that number is projected to exceed 4.5 million by 2026 [52]. Many breast cancer survivors struggle with long-term physical, psychological, and financial scars, even years after their initial treatment has ended [53,54,55]. Breast cancer most often strikes during midlife, a time when women hold multiple responsibilities within the home, workplace, and community. A breast cancer diagnosis impacts not only individual patients but also their families and communities. The total annual cost of breast cancer care in the United States in 2020 was projected to be $20.5 billion [56].

## 4. Comprehensive Cancer Control Plans in the United States

Since 1998, the Centers for Disease Control and Prevention (CDC)’s National Comprehensive Cancer Control Program has supported state-level efforts to bring together coalitions of stakeholders to develop and implement comprehensive strategic plans to prevent and control cancer [57]. Using local data on cancer, all 50 states, the District of Columbia, and several tribes and territories have developed comprehensive cancer control plans that incorporate evidence-based interventions.

CDC priorities for comprehensive cancer control emphasize: the primary prevention of cancer; coordination of early detection and treatment activities; the public health needs of cancer survivors; use of policies, systems, and environmental strategies to guide sustainable cancer control; promotion of health equity as it relates to cancer control; and measurement of outcomes through evaluation (https://www.cdc.gov/cancer/ncccp). Cancer plans include strategies intended to direct collective efforts toward specific and measurable objectives that aim to reduce both cancer incidence and mortality [58]. For breast cancer, plans to date have focused more on secondary prevention (screening and early detection) than on primary prevention.

The National Academy of Sciences, Engineering and Medicine recently convened a committee to develop a national strategy for cancer control in the United States, and the committee’s report was issued in 2019 [59]. Primary cancer prevention, keeping people from developing cancer, was described as the most desirable outcome in cancer control. The committee acknowledged the importance of environmental factors and health behaviors as targets for cancer prevention efforts. However, they also stated that prevention extends beyond public health practice into clinical care. The only prevention interventions mentioned in the report for breast cancer were oral drugs (e.g. tamoxifen) and prophylactic mastectomy in women at high risk for cancer. Because most breast cancers occur among women considered at average risk based on known risk factors [60,61], most breast cancer cases would not be addressed by such clinically-based preventive interventions.

## 5. California’s Comprehensive Cancer Control Plan

In 2002, California received funding from CDC to establish California’s comprehensive cancer control infrastructure, the California Comprehensive Cancer Control Program (CCCP), housed in the California Department of Public Health (CDPH). As part of its comprehensive cancer control efforts, CCCP administers the statewide comprehensive cancer control coalition, the California Dialogue on Cancer (CDOC), which assesses the burden of cancer in the state and coordinates the development and implementation of California’s comprehensive cancer control plan (cancer plan).

California’s first cancer plan was published in 2004 [62], followed by plans for 2011–2015 [63] and 2016–2020 (S. Davis-Patterson, personal communication). The next iteration of California’s cancer plan is currently under development and is expected to begin in 2021, with objectives to be achieved by 2025. Diverse subject matter experts from academia, business, health care organizations, cancer centers, state and local agencies, non-profit organizations, consumer and advocacy groups participated in developing the cancer plan. Development was principally guided by the CDC’s National Comprehensive Cancer Control Program priorities (https://www.cdc.gov/cancer/ncccp).

Breast cancer objectives included in California’s cancer plans have emphasized secondary prevention (through early detection and screening) and better treatment and survivorship outcomes. Objectives have focused on: screening; increasing early stage diagnosis; reducing mortality through research and improved detection methods; tracking women’s breast health care; education for health care professionals, policy makers, and consumers regarding risk reduction; providing educational resources to the community; and improving access to care. In addition, other objectives have addressed the necessity of consistent communication among patients, providers, payers, policy makers, and advocates regarding differences in screening recommendations and shared decision-making.

Since 2002, countless partnerships have been established to assist with the development and implementation of California’s cancer plan. Partners represent a variety of organizations and interest areas, including state and local government, private and nonprofit organizations, health, medical communities, academic institutions, researchers, cancer survivors, caregivers, and advocates. Although CCCP and CDOC facilitate implementation of the plan’s strategies, partnerships are essential in achieving the goals and objectives of the plan. Ultimately, implementation of the cancer plan is the responsibility of all cancer control stakeholders in California.

Strategies out of the cancer plan that are implemented specifically by CCCP and CDOC are identified based on coalition priorities, program, and coalition capacity and resources, and CDC priorities. CDOC establishes workgroups to specifically address these priorities. Although CDOC has not facilitated interventions through an established breast cancer workgroup, other CDOC workgroups have addressed breast cancer indirectly through other efforts, such as educational material development and community events related to screening awareness (for all recommended cancer screening tests). Other efforts include working with California’s breast and cervical cancer early detection program, the Every Woman Counts program, on screening awareness activities.

Comprehensive cancer control relies on collaborations and partners pooling resources to implement cancer plan objectives, but consistent resources are not always available for some efforts. However, dedicated and passionate stakeholders continue to work with CCCP and CDOC to develop objectives to guide the work of reducing the breast cancer burden in California. In addition, stakeholders facilitate efforts statewide that contribute to the achievement of California’s breast cancer objectives related to screening and early detection.

## 6. Breast Cancer Primary Prevention Plan for California

Given the lack of progress in breast cancer prevention, the California Breast Cancer Research Program (CBCRP) thought the time was right to apply current scientific knowledge about breast cancer to the primary prevention of breast cancer at the population level. CBCRP is separate from the CDOC and receives no funds from the CCCP. To turn the tide of breast cancer in the state, CBCRP supported the development of a comprehensive, primary prevention plan for breast cancer in California. CBCRP funded a team at Breast Cancer Prevention Partners (BCPP) to convene leaders in advocacy, policy, and research related to breast cancer prevention from across the state of California. The objective was a strategic plan to direct collective efforts toward specific and measurable objectives to reduce the incidence of breast cancer.

BCPP began with a vision for breast cancer prevention that was unique in two important ways. First, they focused on primary prevention. The BCPP vision broke from the traditional cancer control continuum that spans from prevention through treatment and end of life: the sole focus was stopping breast cancer before it starts. By targeting primary prevention, BCPP broadened the prevention lens from clinical settings to the communities where people live, work and play.

Second, BCPP considered factors that could be modified through policy, environment and system changes to reduce breast cancer incidence at a population level. Many sources list modifiable risk factors for breast cancer that, at least in theory, are amenable to an individual’s choice, such as physical activity, breastfeeding and alcohol consumption [10]. BCPP moved the focus upstream, to identify interventions at the community level to tackle systemic barriers to health and obstacles to adopting health behaviors. 

BCPP aimed to develop a comprehensive policy agenda for breast cancer prevention that was both effective and practical. The approach touched on all levels of the health impact pyramid, from education at the top to the bottom rungs of changing the context and socioeconomic factors, where the population impact is greatest [64]. The agenda also considered risk factors at all stages of the lifespan. Although the plan was for the state of California, BCPP hoped to develop a model that other states could follow to develop their own breast cancer prevention plans or that could be adapted for use at the national level.

At the outset, BCPP articulated a commitment to use the best science and policy ideas, and to actively seek out the perspective and input of people who had been underrepresented in research and policy discussions. With input from the plan’s diverse advisory committee, BCPP laid out five guiding principles, then challenged this advisory committee and community allies to hold them accountable for following these guiding principles throughout the development of the plan. The guiding principles [65] were as follows:

1. Breast cancer is a societal issue. Reducing risk requires systemic change.
2. In order to create a healthy society, we must address the multigenerational trauma from and violence of discrimination, racism, income inequality and disparities in power and access.
3. Community wisdom is a valuable source of information and often highlights areas scientific research has not yet investigated.
4. Breast cancer risk is multi-factorial. Interventions to reduce risk should also be multi-factorial.
5. We do not need 100% certainty to act.

Given the complexity of creating a comprehensive breast cancer primary prevention plan, BCPP sought input from a wide range of sources. The process of identifying the best information from these various sources was done simultaneously. An advisory committee of academics, community and nonprofit leaders, and government agency staff was convened to represent the depth and breadth of knowledge in the wide range of breast cancer risk factors and potential interventions.

BCPP used seven foundational documents [7,8,33,66,67,68,69], developed by committees and task forces that relied on peer-reviewed literature, to identify a preliminary list of breast cancer risk and protective factors. The list was presented to the advisory committee and community participants for feedback, resulting in a list of 23 risk and protective factors (Table 1). To ensure that the plan reflected the current state of the science, scoping reviews were conducted to identify new information since the release of the foundational documents. Nine on-line study group sessions (1.5 hour-long webinars) were held on topics selected by the advisory committee, to explore complexities in the science and potential interventions. The final plan provides a brief summary of the state of the scientific evidence, with references to the scientific literature, for each risk and protective factor.

To identify potential preventive interventions, BCPP searched authoritative sources of evidence reviews and recommendations related to the 23 risk and protective factors, including the Cochrane Library (https://www.cochranelibrary.com/), the U.S. Preventive Services Task Force (https://www.uspreventiveservicestaskforce.org/), NICE, UK National Institute for Health and Care Excellence (https://www.nice.org.uk/), Research-tested Intervention Programs (RTIPs) from the National Cancer Institute (https://rtips.cancer.gov/rtips/index.do), and the U.S. Community Preventive Services Task Force (https://www.thecommunityguide.org/). Other sources included: Pubmed and potential interventions highlighted on the internet by nonprofit organizations; local, regional, and state governments; school districts; and community groups.

To incorporate community perspectives into the plan, BCPP undertook a multiple-step process of community engagement and relationship building. Over the course of the project, BCPP met with community members and groups at listening sessions, each lasting 2.5 to 4 hours, in 11 communities throughout California. At the completion of the listening sessions tour, synthesized notes were distributed to all 125 participants in the listening sessions, and a webinar for participants and interested stakeholders summarized the themes from the sessions. Participants were invited to provide comments on the information presented. In addition, three community leaders served on the advisory committee, and a core group of community representatives was convened in-person for two days to provide in-depth verbal and written feedback on a draft of key sections of the report.

Data and information collected from the various sources identified 450 intervention needs or ideas to inform policy, systems, or environmental approaches that could be taken by the state, cities, school districts, counties, private companies, and other institutions. The intervention ideas were evaluated according to the strength of evidence, the needs identified by consulted community members, and alignment with the guiding principles. The recommendations were finalized after numerous rounds of review that provided opportunities for input from many stakeholders. The plan seeks to serve as a catalyst to spark new approaches, new partnerships, and new ideas, and as a jumping off point for creating new solutions to reduce breast cancer and other health problems. The final plan is expected to be available in spring 2020 on the BCPP website (www.bcpp.org).

## 7. Conclusions

The adverse consequences of breast cancer to society will continue to grow, unless more is done to prevent breast cancer incidence. The new prevention approach taken by BCPP embraces a more expansive mind frame for prevention and maps a pathway for the future, providing a menu of options that can be taken at the state, county, or local level. The priorities in the breast cancer primary prevention plan for California complement, not conflict, with California’s comprehensive cancer control plan. The prevention plan identifies new opportunities to collaborate with members of California’s cancer coalition and relevant stakeholders that are implementing similar interventions as part of the state’s cancer control plan.

The search for knowledge about breast cancer causes and prevention can occur simultaneously with actions based on current knowledge. Identified gaps in data in the prevention plan can point to priority areas for further research and technology development. Where the evidence base is lacking on the effectiveness of intervention strategies, innovative approaches informed by the science and community input can be developed and evaluated. Prevention strategies can be compared to identify those likely to have the greatest impact with the lowest cost. Community perspectives can inform the implementation of prevention strategies to reduce inequities and unintended consequences. Additional information on the dimensions of exposure to a risk factor in specific communities, including prevalence, intensity, frequency, and timing over the life course, can be used to set priorities.

The prevention strategies identified in the breast cancer primary prevention plan for California have the potential to reduce the number of incident breast cancer cases in the future, consistent with national objectives to reduce the human cost of cancer. In the more immediate future, the prevalence of comorbidities common among older breast cancer survivors could be reduced [70], thus enhancing their health and wellness. Many of the cancer risk factors addressed in the plan contribute to other cancers and other chronic diseases, and communities with a lower risk of breast cancer could experience other health benefits. Breast cancer prevention can serve as a bellwether for community health.

Through the development of the breast cancer primary prevention plan for California, the CBCRP and BCPP have launched an expanded dialogue within California on the systemic factors that contribute to breast cancer incidence and poor health. This expanded dialogue has engaged community representatives, a transdisciplinary group of scientists, and a broad range of partners and stakeholders. The structured, innovative approach used by BCPP to integrate scientific evidence with community perspectives provides a model for other states to consider, to potentially change the future trajectory of breast cancer incidence in the United States.

## Figures and Tables

**Table 1:** Inventory of presumptive breast cancer risk and protective factors examined by Breast Cancer Prevention Partners (BCPP).

Alcohol consumption	Ambient noise
Body weight over the life span	Breast density
Breastfeeding and lactation	Chemicals (overview) and consumer products
Inflammation	Ionizing radiation
Light at night	Menarche and menopause
Microbiome	Non-ionizing radiation
Nutrition and dietary factors	Occupational factors
Parity and age at first birth	Pharmaceutical hormone use
Physical activity	Place-based chemicals
Race, power and inequities	Social and built environment
Stress	Tobacco
Vitamin D	

Note: BCPP used seven foundational documents [7,8,33,66,67,68,69], to identify presumptive breast cancer risk and protective factors. The preliminary list was presented to the advisory committee and community participants for feedback, resulting in this final list. The strength of evidence, extent of exposure, and community-based intervention strategies vary by factor.
